# Measurement of Social Cognition in Amyotrophic Lateral Sclerosis: A Population Based Study

**DOI:** 10.1371/journal.pone.0160850

**Published:** 2016-08-24

**Authors:** Tom Burke, Marta Pinto-Grau, Katie Lonergan, Marwa Elamin, Peter Bede, Emmet Costello, Orla Hardiman, Niall Pender

**Affiliations:** 1 Department of Psychology, Beaumont Hospital, Dublin, 9, Ireland; 2 Academic Unit of Neurology, Trinity Biomedical Sciences Institute, Dublin, 2, Ireland; 3 Department of Neurology, Beaumont Hospital, Dublin, 9, Ireland; 4 Royal College of Surgeons in Ireland, Dublin, 2, Ireland; Centre of Genomic & Post Genomics, ITALY

## Abstract

Background: Amyotrophic lateral sclerosis (ALS) is a rapidly progressive neurodegenerative disease. Executive dysfunction is common in patients with ALS, with up to 50% of patients performing within an impaired range. There is evidence that social cognitive deficits associated with ALS are a function of deficits in executive function. The ‘Reading the Mind in the Eyes’ Test is a recognized test of social cognitive function, although the reliability of this instrument remains to be established. Methodology: Patients with ALS (n = 106), and age and IQ matched controls (n = 50) were recruited and asked to perform the Reading the Mind in the Eyes Test as part of an on-going population-based study of cognitive function. ALS patients were sub-stratified based on the presence, and/or extent of executive dysfunction. Results: Cronbach’s Alpha of .73 was observed, indicating good reliability on this measure. Split-half reliability analysis further confirms these findings (*p* = 0.826). The Reading the Mind in the Eyes test had excellent psychometric properties when discriminating between ALS patients who are cognitively intact, and those who have executive impairment, with an overall medium difficulty. There was a large magnitude significant difference between patients and controls (*p*< 0.001; η^2^ = .19). Post-hoc analysis revealed that controls performed significantly higher than patients with executive impairment (*p*< 0.001), and patients with single executive deficits (*p* = 0.002). Conclusion: Executive dysfunction impacts on social cognitive performance. This study contributes not only to the psychometric knowledge of this measure, but also to the usability, efficacy, and reliability of social cognitive assessment in ALS. Using population-specific normative data, we confirm the Reading the Mind in the Eyes Test is a reliable measure of social cognitive processes in ALS.

## Introduction

Amyotrophic lateral sclerosis (ALS) is a rapidly progressive and fatal neurodegenerative disorder. Cognitive impairment, specifically executive dysfunction is common in patients with ALS, with up to 50% of patients performing within an impaired range [1]. Behaviour changes are also known to occur in ALS, with apathy as the most commonly reported symptom [2]. Recently, an in-depth study of the social cognitive changes in ALS found that performance in social cognitive function varies based on the measure used [3].

Social cognition can be defined as the ability to represent and attribute affective and cognitive mental states [4]. It integrates cognitive processes such as the ability to follow eye-gaze, share attention, recognize emotion, and to distinguish between self and other [5]. Initial social cognition research focused on disorders where social skills were implicated as a core feature [6, 7]. However, in recent years the relationship between neurodegeneration and social cognitive processes has gained much attention [8] and assessments of neuropsychological and social cognitive performance are more routine in neurologic conditions where muscular atrophy is the dominant feature [9].

Performance on measures of social cognition in ALS has been associated with fronto-striatial dysfunction. ALS-related executive dysfunction has been reported to be the main predictor of social cognition performance when compared with demographic variables, behaviour, mood and personality [10]. Social cognitive deficits are also a well-recognised feature of Frontotemporal dementia [11] (FTD). ALS with a comorbid FTD is known to occur in 10–15% of patients [12], with a strong clinical and pathological overlap between these conditions [13]. Cognitive features, such as impaired social cognition of FTD may therefore be evident in people with ALS, whom do not meet the criteria for a comorbid dementia syndrome.

A commonly used social cognition task in neurodegenerative research is the Reading the Mind in the Eyes Test [3, 10, 14]. The Reading the Mind in the Eyes Test [6] (RMET) comprises photographs of eye regions of human faces where participants are required to infer their mental/emotional state given four possible choices. The RMET was chosen as a measure of affective social cognition within this cohort, as individuals can respond verbally or by pointing. Although the RMET was not specifically developed for use with an ALS cohort, it accounts for potential bulbar/spinal motor disability. The RMET has also been used with a wide variety of clinical populations with neurological conditions, including patients with ALS [15], Kennedy’s disease [9], Huntington’s disease [16], Lewy body dementia [17], Parkinson’s Disease [18], Epilepsy [19], as well as psychiatric populations [20], children [21, 22] and healthy cohorts [23–25]. However the reliability of the measure has been subject to criticism. Notwithstanding, recent evidence suggests that the RMET has good validity [26], and is unaffected by the test-taker’s gender [27] in some but not all instances. Conflicting reports of reliability may be due to cultural differences in interpreting the stimuli.

The aim of our study was to investigate the reliability of the RMET using a control cohort, to sub-stratify ALS patients based on cognitive categorisation criteria [28] and to compare cohorts of multi-domain executive deficits (cognitively impaired), single executive deficits, and no cognitive abnormalities detectable on testing. We further investigated the difficulty and discrimination coefficients associated with this measure in an Irish population-based cohort of ALS patients and healthy controls.

## Materials and Methods

### Participants

A total of 108 population based ALS patients were included in this study. The research design, case ascertainment, procedure, and information on the population-based register have been previously reported in detail [1, 14, 29, 30]. ALS patients were classified into groups specific to cognitive status, based on cognitive domain-based criteria. This methodology has previously been reported in detail [29], and is based on internationally accepted consensus criteria for cognitive impairment [30]. This method further compliments these criteria as a more stringent cut-off was used to define abnormality. Using these criteria, patients were sub-stratified based on whether they had ‘no cognitive abnormalities’ (n = 70), a ‘single executive deficit’ (n = 19), or ‘multi-executive deficits/cognitively impaired’ (n = 19).

Due to ALS patient stratification based on severity of executive impairment, we completed a priori power analyses to ensure our findings with *F* statistics would be robust with smaller groups. A standard alpha error probability of .05 was employed, with a 1-β power of .8 recruited for a 4 group design. This yielded a non-centrality parameter λ of 12.16, critical F at 2.73, and actual power of .82, which required a minimum of 19 per group.

Inclusion criteria for this research were a diagnosis of a possible, probable or definite ALS based on the El Escorial criteria [31], with supported neurophysiology for diagnostic accuracy. Exclusion criteria included the presence of co-morbid neurological and/or psychiatric condition such as stroke, psychosis, traumatic brain injury, or an active or positive history of chronic substance abuse. Clinic- and home-based assessments were used to gather clinical and neuropsychological data. In addition, 50 healthy age- and education-matched controls were recruited considering the aforementioned exclusion criteria.

The Medical Research Ethics Committee of Beaumont Hospital, Dublin, Ireland, has approved this study. Informed consent was obtained from all participants in this study.

### Cognitive Measures

The Reading the Mind in the Eyes Task is a 36-item assessment where photographs of eye regions are presented, and participants are required to infer the mental/emotional from four choices e.g., terrified, upset, bored, irritated.

Participants were also assessed using a battery of standardized neuropsychological measures and cognitively categorised as noted above [29]. Variables of interest were extracted for the purpose of this study, which included the Wechsler Test of Adult Reading [32] (WTAR), a measure of premorbid function yielding a predicted Full Scale IQ (pFSIQ). Ravens Progressive Coloured Matrices [33] investigated current function through verbal/non-verbal trials.

### Ethical Considerations

The Beaumont Hospital Medical Research Ethics Committee approved this study. All procedures were conducted in accordance to the principles expressed in the Declaration of Helsinki. Written informed consent was obtained from all participants in this study.

### Statistical Methods

Demographic characteristics between ALS patients and controls were analysed using independent samples t-tests (age and years of education), with *χ*^2^ used to test dichotomised variables. ANOVA were used to compare multiple dependent variables. The threshold for statistical significance was set at p<0.05 as per our power calculations. All statistical analyses were conducted using SPSS, Version 22.0.

#### Reliability and Validity

Classification for good internal consistency, using Cronbach’s alpha, remains at the internationally accepted value of >.70. Split half reliability was also assessed using Spearman-Brown coefficient for equal length measures, to compliment analyses of internal consistency.

#### Item difficulty

We computed the difficulty and discrimination coefficient of each item in order to test if it yielded the necessary degree of reliability and validity [34]. This methodology has previously been employed when investigating the psychometric properties of a translated version of the RMET [35]. We infer that a reliable and valid item should be able to appropriately distinguish between those with a higher total score and those with a lower total score on the RMET. We defined the higher score and lower score groups as the upper 27% and the lower 27% of participants, according to their total score on RMET consistent with previous studies [36].

### Difficulty Coefficient

The proportion of participants who answer a test item correctly relative to the test taking population measures the difficulty of an item. The higher this proportion is, the lower the item’s difficulty. To calculate the difficulty of an individual test item, the number of participants who answered the item correctly (from both the upper and lower proportions) is divided by the total number of participants who took the test. It is represented as the following formula:

Pi=Ai/Ni×100

whereby Pi = difficulty index of item; Ai = number of correct answers in upper 27% added to the number of correct answers in the lower 27%; Ni = the sum of total test takers in the upper 27% and lower 27% groups.

Difficulty levels are classified in the following way [34]: very difficult (Pi < 30%); moderately difficult (31%–50%); medium difficulty (51%–70%); moderately easy (71% - 90%); and very easy (Pi > 90%). The ideal distribution of difficulty follows the basic principles of normal distribution.

#### Discrimination Coefficient

Items of a valid and reliable test must be able to appropriately differentiate between participants who are relatively strong from those who are relatively weak; otherwise, the test is lacking sensitivity and specificity. Through the use of healthy controls, we investigated the RMET’s overall discrimination coefficient. We then also applied this methodology to our ALS cohort whereby the proportion of patients who scored in the upper range reflects patients with a ‘no cognitive abnormality detected’ status, and the proportion of patients who scored in the lower group represents the ‘multi executive deficits’ apparent on testing group. Using this method we calculate the ability of this task to discriminate between ALS patients who are cognitively intact, and those who are cognitively impaired based on internationally accepted criteria.

It is calculated through the following formula:

Di=(Pu−Pl)×100

whereby Di: index of discrimination of item; Pu: the proportion of those in the upper 27% group who correctly scored on the item relative to the sum of test takers; Pl: the proportion of those in the lower 27% group who correctly scored on the item relative to the sum of test takers.

The following guidelines have been published [30] to interpret the Di values: D ≥40 = Excellent; 39–30 = Good; 29–20 = Mediocre; 19–0 = Poor; D < -1 = Worst.

## Results

### Control Demographics and Psychometric Reliability

Control participants at the time of assessment were 61.4 years SD± 9.1, with 12.7 years of education SD±2.9. This cohort consisted of 30 males (60%). Prior to conducting reliability analyses, we investigated whether participant gender was equally distributed within our control cohort. The distribution of gender within the control cohort was found to be homogenous (*p* = 0.203). We investigated whether there was a significant difference in performance based on gender, with a 2-tailed hypothesis. With equal variance assumed, we report that there was no difference identified on the score of the RMET, based on gender stratification (*p* = 0.113).

We proceeded to conduct reliability statistics, and considering the full 36-items of the RMET, a Cronbach’s Alpha of .73 was observed. The reliability of each individual test item, and how it correlates to the overall measure can be seen in Table 1. Split-half reliability analyses were conducted using Spearman-Brown Coefficient, and equal length statistics confirm the reliability of this measure (*p* = 0.826).

**Table 1:** Itemised Reliability Statistics.

	Scale Mean if Item Deleted	Scale Variance if Item Deleted	Corrected Item-Total Correlation	Cronbach's Alpha if Item Deleted
Playful	24.38	22.48	.340	.712
Upset	24.64	20.92	.566	.694
Desire	24.38	22.77	.252	.716
Insisting	24.74	23.38	.021	.730
Worried	24.62	21.54	.428	.704
Fantasizing	24.62	22.15	.290	.713
Uneasy	24.34	23.73	-.032	.727
Despondent	24.42	23.10	.129	.722
Preoccupied	24.44	22.86	.184	.719
Cautious	24.42	23.96	-.099	.733
Regretful	24.58	21.59	.430	.704
Sceptical	24.40	22.08	.436	.707
Anticipating	24.44	22.74	.216	.717
Accusing	24.44	22.29	.336	.711
Contemplative	24.48	22.25	.318	.712
Thoughtful	24.48	22.25	.318	.712
Doubtful	24.50	23.27	.062	.726
Decisive	24.74	23.70	-.046	.734
Tentative	24.62	23.71	-.045	.733
Friendly	24.46	22.13	.363	.709
Fantasizing	24.66	22.43	.223	.717
Preoccupied	24.34	22.55	.379	.711
Defiant	24.62	23.26	.050	.728
Pensive	24.66	22.10	.295	.712
Interested	24.58	23.14	.079	.726
Hostile	24.80	21.59	.406	.705
Cautious	24.62	21.58	.419	.704
Interested	24.44	21.96	.425	.706
Reflective	24.44	24.12	-.140	.735
Flirtatious	24.36	22.11	.491	.706
Confident	24.82	22.96	.109	.724
Serious	24.72	22.36	.233	.716
Concerned	24.56	22.21	.294	.713
Distrustful	24.60	22.61	.193	.719
Nervous	24.60	23.02	.104	.724
Suspicious	24.44	23.06	.130	.722

----Split-half reliability section break

Each item was analysed to investigate a difficulty coefficient (Pi) and was classified accordingly. A discrimination coefficient (Di) and classification were employed to investigate whether the individual items are accurate at discriminating between controls who performed well compared to those with a low score on the test. Overall the RMET is reported to be of medium difficulty (68.8%), with good psychometric properties for discriminating healthy controls based on performance (Di = 31.7). The breakdown of individualised items can be found in Table 2.

**Table 2:** Control Difficulty and Discriminatory Performance.

Q	Pi	Pi Classification	Di	Di Classification
1	75.0%	Mod. Easy	35.80	Good
2	46.4%	Mod. Difficulty	78.60	Excellent
3	82.1%	Mod. Easy	21.50	Mediocre
4	60.7%	Medium	21.40	Mediocre
5	53.6%	Medium	64.50	Excellent
6	67.9%	Medium	50	Excellent
7	96.4%	Very Easy	7.10	Poor
8	75.0%	Mod. Easy	7.20	Poor
9	82.1%	Mod. Easy	35.70	Good
10	85.0%	Mod. Easy	-14.30	Worst
11	60.7%	Medium	64.30	Excellent
12	78.6%	Mod. Easy	42.90	Excellent
13	75.0%	Mod. Easy	35.80	Good
14	78.6%	Mod. Easy	28.60	Mediocre
15	71.4%	Mod. Easy	42.90	Excellent
16	71.4%	Mod. Easy	28.60	Mediocre
17	71.4%	Mod. Easy	14.30	Poor
18	50.0%	Mod. Difficulty	14.20	Poor
19	60.7%	Medium	7.20	Poor
20	71.4%	Mod. Easy	42.90	Excellent
21	60.7%	Medium	35.70	Good
22	85.7%	Mod. Easy	28.60	Mediocre
23	82.1%	Mod. Easy	7.10	Poor
24	50.0%	Mod. Difficulty	42.80	Excellent
25	67.9%	Medium	35.70	Good
26	35.7%	Mod. Difficulty	57.20	Excellent
27	57.1%	Medium	57.10	Excellent
28	75.0%	Mod. Easy	50	Excellent
29	78.6%	Mod. Easy	0	Poor
30	82.1%	Mod. Easy	35.70	Good
31	46.4%	Mod. Difficulty	21.40	Mediocre
32	57.1%	Medium	42.90	Excellent
33	67.9%	Medium	35.70	Good
34	67.9%	Medium	21.60	Mediocre
35	64.3%	Medium	28.60	Mediocre
36	85.7%	Mod. Easy	14.30	Poor
*X*	68.82%	Medium	31.77	Good

Pi = Item’s difficulty

Di = Item's ability to differentiate between those who are high and low

### ALS Demographics and Comparative Performance

Considering the reliability of the measure, we investigated the serial performance of controls on each item, as well as the scores of ALS patients. Patients at the time of assessment were 60.4 years SD± 10.8, with 12.8 years of education SD±3.3. ALS patients were mostly spinal onset (73.5%) and male (71.6%).

**Fig 1:**
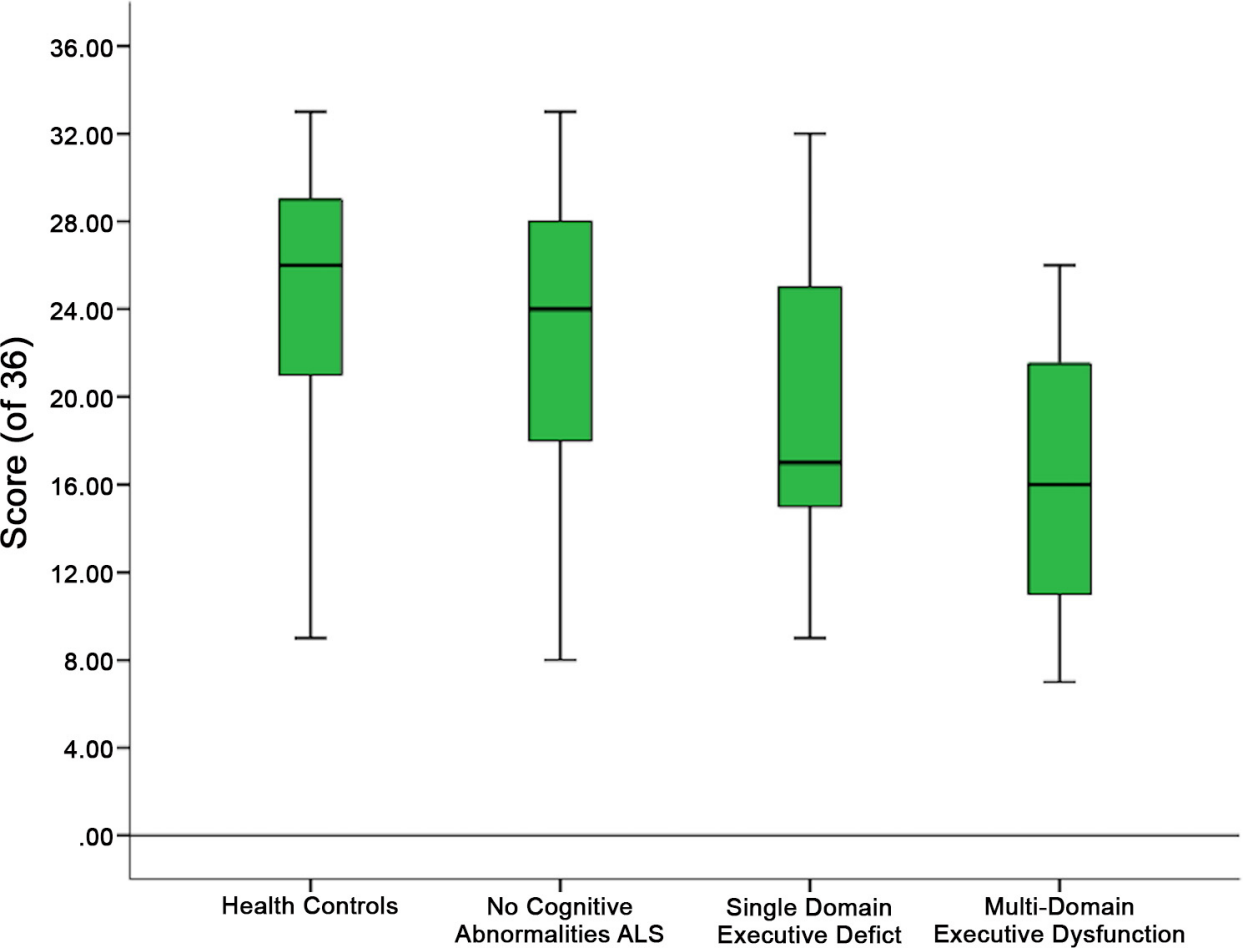
Distribution of Scores on the RMET. Patient and Control Performance on the Reading the Mind in the Eyes Test, stratified by cognitive categorisation.

Fig 1 illustrates that control performance was highest (n = 50; *X* = 25.24 ± 4.87), followed by ALS patients with no cognitive abnormalities (n = 70; *X* = 22.9 ±6.46), followed by patients with a single executive impairment (n = 19; *X* = 19.42 ±6.71), and patients with multi-executive impairments had the lowest performance (n = 19; *X* = 16.15 ±6.22). Fig 1 highlights cumulative performance of each group using the RMET with evident decline associated with grouping based on executive performance.

Individual patient groups’ performance was compared to healthy controls, with groups matched for age at assessment (*p* = 0.622), and years of education (*p* = 0.940). Homogeneity of variance was satisfied (*p* = 0.115), and an ANOVA was conducted as per our power calculation. There was significant difference with large magnitude reported from the ANOVA (*p*< 0.001; η^2^ = .19).

To elucidate this further, a post-hoc analysis was employed. Controls performed significantly higher than patients with executive impairment (*p* = 0.001), and patients with single executive deficits (*p* = 0.002). Controls did not significantly differ from ALS patients with no cognitive abnormalities (*p* = 0.157). By contrast, ALS patients without cognitive deficits did not differ from ALS patients with single executive deficits (*p* = 0.118), yet did perform significantly better than ALS patients with multi-domain executive impairment (*p* = 0.001). Interestingly, ALS patients with single executive deficits did not differ from the patients with multi-domain executive impairment (*p* = 0.341).

#### Psychometrics in ALS patients

Itemised group performance can be seen in Table 3, where scoring patterns remain consistent based on executive grouping. The Reading the Mind in the Eyes test appears to have ‘Excellent’ psychometric properties when discriminating between patients who are cognitively intact, and those who have executive impairment (Di = 43.5), with an overall ‘Medium’ difficulty (54.7%). Individual item difficulty and discrimination for ALS patients are presented in Table 4.

**Table 3:** Individual Item Performance (% correct).

Item	Healthy Controls (N = 50)	Cognitively Intact ALS (N = 70)	Single Cognitive Impairment (N = 19)	Multi-Executive Impairment (N = 19)
1	86	74	63	42
2	60	63	57	52
3	86	71	68	42
4	50	60	47	36
5	62	64	57	57
6	62	74	42	52
7	90	70	57	47
8	82	74	68	57
9	80	71	42	42
10	82	59	68	47
11	66	70	73	47
12	84	69	47	26
13	80	66	42	36
14	80	70	57	36
15	76	69	31	26
16	76	77	78	52
17	74	63	63	52
18	50	53	47	26
19	62	53	47	36
20	78	73	68	73
21	58	53	31	42
22	90	76	52	52
23	62	57	47	21
24	58	61	31	26
25	66	60	63	63
26	44	49	52	36
27	62	59	47	52
28	80	71	52	36
29	80	63	78	36
30	88	69	52	47
31	42	43	36	26
32	52	47	47	52
33	68	54	47	57
34	64	56	36	42
35	64	60	57	57
36	80	70	73	68

**Table 4:** ALS Difficulty and Discrimination Performance.

Q	Pi	Pi classification	Di	Di Classification
1	53.12%	Medium	56.20	Excellent
2	56.25%	Medium	37.50	Good
3	52.77%	Medium	56.30	Excellent
4	50%	Mod. Difficulty	50	Excellent
5	56.25%	Medium	12.50	Poor
6	56.25%	Medium	62.50	Excellent
7	71.87%	Mod. Easy	31.30	Good
8	56.25%	Medium	62.50	Excellent
9	50%	Mod. Difficulty	75	Excellent
10	46.87%	Medium	6.30	Poor
11	68.75%	Medium	25	Mediocre
12	50%	Mod. Difficulty	37.50	Good
13	56.25%	Medium	62.50	Excellent
14	56.25%	Medium	50.20	Excellent
15	43.75%	Mod. Difficulty	87.50	Excellent
16	65.62%	Medium	31.20	Good
17	56.25%	Medium	37.50	Good
18	40.62%	Mod. Difficulty	31.20	Good
19	46.87%	Mod. Difficulty	43.70	Excellent
20	68.75%	Medium	37.50	Good
21	46.87%	Mod. Difficulty	56.30	Excellent
22	56.25%	Medium	87.50	Excellent
23	56.25%	Medium	50	Excellent
24	40.62%	Mod. Difficulty	68.80	Excellent
25	56.25%	Medium	37.50	Good
26	53.12%	Medium	31.20	Good
27	59.37%	Medium	43.70	Excellent
28	59.37%	Medium	43.70	Excellent
29	56.25%	Medium	37.50	Good
30	62.50%	Medium	37.50	Good
31	37.50%	Mod. Difficulty	0	Poor
32	43.75%	Mod. Difficulty	37.50	Good
33	46.87%	Mod. Difficulty	43.70	Excellent
34	56.25%	Medium	37.50	Good
35	68.75%	Medium	25	Mediocre
36	68.75%	Medium	37.50	Good
Ave	54.76%	Medium	43.58	Excellent

Pi = Item’s difficulty; Di = Item's ability to differentiate between those who are high and low

## Discussion

Our primary aim was to investigate whether the Reading the Mind in the Eyes Test (RMET) is a reliable measure. Secondary aims included addressing its efficacy and validity as a measure against a patient population, where executive dysfunction is categorised with high accuracy. These data suggest that the RMET is a reliable measure of social cognition for use with healthy and ALS cohorts (Cronbach’s alpha .73; Split-half reliability *p* = .826). Our data further support previous work [27] that the RMET score is not affected by gender (*p* = 0.113).

Overall the RMET is reported to be of medium difficulty (68.8%), with good psychometric properties for discriminating between controls that perform in the upper and lower ranges (Di = 31.7). This analysis was applied to an ALS cohort, as a total group. The lower 27% of the patient group had a range of executive deficits, and the upper 27% were cognitively intact. This allowed us to infer whether this measure is useful in dichotomising executive dysfunction in ALS. These data suggest that this measure has ‘Excellent’ psychometric properties (Di = 43.5) for discriminating between patients with cognitive impairment and those who are cognitively intact, with an overall ‘Medium’ difficulty (54.7%).

Consistent with previous literature, there was significant difference with large magnitude reported between patient groups and the control cohort (*p*< 0.001; η^2^ = .19). Post-hoc analysis revealed that controls performed significantly higher than patients with executive impairment (*p* = 0.001), and patients with single executive deficits (*p* = 0.002) but not cognitively intact ALS patients (*p* = 0.157). Of interest were the scores of ALS patients with single executive deficits, as they did not differ from the patients with multi-domain executive impairment (*p* = 0.341) or cognitively intact ALS patients (p = 0.118). This pattern of performance demonstrates that ALS patients without cognitive abnormalities are similar to controls, and to those with single domain cognitive impairment (See Fig 1). Patterns of social cognitive deficits such as these are consistent with both the subtle deficits of executive function, which may present early in the disease, as well as the more severe impairment known to occur in some cases. The lack of significant difference in performance on the RMET between groups i.e., no cognitive abnormalities and single executive impairment, could be attributed to difficulties in detecting subtle cognitive impairment in this cognitive domain.

This study has limitations. Given the lower scoring profile of cognitively intact ALS patients, longitudinal follow-up would be advantageous. This point is further generalised to the other ALS cohorts to quantify the rate that social cognitive processes decline alongside the disease trajectory, with respect to executive dysfunction. Given the relevance of these findings in relation to executive function, future research could investigate the impact of social cognitive deficits on patient survival, the relationship of social cognitive deficits to behavioural presentations, and the extent to which patients differ on test performance considering genetic expansions known to implicate cognitive function i.e., C9orf72. Future studies could investigate the relationship between social cognitive performance and behavioural features associated with ALS; could determine whether social cognitive decline has a negative impact on caregiver burden in ALS; and whether there are additional negative implications on the psychological wellbeing of patients and caregivers.

In conclusion, our data confirm the Reading the Mind in the Eyes Test is a reliable measure of social cognitive processes, and illustrate how executive dysfunction mediates performance on this measure. These data provide clinically relevant normative data for use in clinical and research settings. Moreover, by stratifying by level of executive impairment here, these data provide a useful reference point in terms of severity of social cognitive deficit. Clinically these data support the routine inclusion of social cognitive measures in standardised batteries of neuropsychological tests. Furthermore, considering the RMET as a valid measure of social cognition for use with ALS patients, their overall performance on this measure should be considering in light of ALS-FTD comorbidity.

## References

[1] Elamin M, Phukan J, Bede P, Jordan N, Byrne S, Pender N, et al Executive Dysfunction is a negative prognostic indicator in patients with ALS without dementia. Neurology 2012;76:1263–1289. 21464431 10.1212/WNL.0b013e318214359f

[2] Burke T, Elamin M., Galvin M., Hardiman O., & Pender N. (2015). Caregiver burden in amyotrophic lateral sclerosis: a cross sectional investigation of predictors. J Neurol. 10.1007/s00415-015-7746-z 25904206

[3] Giradi A, MacPherson SE, Abrahams S. Deficits in Emotional and Social Cognition in Amyotrophic Lateral Sclerosis. Neuropsychology 2011;25(1):53–65. doi: 10.1037/a0020357 20919762 10.1037/a0020357

[4] Abu-Akel A, Shamay-Tsoory S. Neuroanatomical and neurochemical bases of theory of mind. Neuropsychologia 2011;49:2971–84 doi: 10.1016/j.neuropsychologia.2011.07.012 21803062 10.1016/j.neuropsychologia.2011.07.012

[5] Adolphs R. The social brain: neural basis of social knowledge. Annu Rev Psychol 2009;60:693–716. doi: 10.1146/annurev.psych.60.110707.163514 18771388 10.1146/annurev.psych.60.110707.163514 PMC2588649

[6] Baron-Cohen S, Wheelwright S, Hill J, Raste Y, Plumb I. The “Reading the Mind in the Eyes” Test revised version: a study with normal adults, and adults with Asperger syndrome or high-functioning autism. J Child Psychol Psychiatry 2001;42:241–51. 11280420

[7] Segura M, Pedreño C, Obiols J, Taurines R, Pàmias M, Grünblatt E, et al Neurotrophin blood-based gene expression and social cognition analysis in patients with autism spectrum disorder. Neurogenetics 2015;6:123–131 doi: 10.1007/s10048-014-0434-9 10.1007/s10048-014-0434-9 25535174

[8] Cavallo M, Adenzato M, MacPherson SE, Karwig G, Enrici I, Abrahams S. Evidence of Social Understanding Impairment in Patients with Amyotrophic Lateral Sclerosis. PLoS one. 2011;6(10):e25948 doi: 10.1371/journal.pone.0025948 21998727 10.1371/journal.pone.0025948 PMC3187828

[9] Di Rosa E, Soraru G, Kleinbub JR, Calvo V, Vallesi A, Querin G, et al Theory of mind, empathy and neuropsychological functioning in X-linked Spinal and Bulbar Muscular Atrophy: a controlled study of 20 patients. Journal of neurology. 2015 2 1;262(2):394–401. doi: 10.1007/s00415-014-7567-5 25408365 10.1007/s00415-014-7567-5

[10] Watermeyer TJ, Brown RG, Sidle KCL, Oliver DJ, Allen C, Karlsson J, et al Executive dysfunction predicts social cognition impairment in ALS. J Neurol 2015; doi: 10.1007/s00415-015-7761-0 10.1007/s00415-015-7761-0 25957636

[11] Bertoux M, de Souza LC, O’Callaghan C, Greve A, Sarazin M, Dubois B, et al Social Cognition Deficits: The Key to Discriminate Behavioral Variant Frontotemporal Dementia from Alzheimer’s Disease Regardless of Amnesia? Journal of Alzheimer's Disease. 2015 11 20;49(4):1065–74. doi: 10.3233/JAD-150686 26756325 10.3233/JAD-150686

[12] Byrne S, Elamin M, Bede P, Shatunov A, Walsh C, Corr B, et al Cognitive and clinical characteristics of patients with amyotrophic lateral sclerosis carrying a C9orf72 repeat expansion: a population-based cohort study. The Lancet Neurology. 2012 3 31;11(3):232–40. doi: 10.1016/S1474-4422(12)70014-5 22305801 10.1016/S1474-4422(12)70014-5 PMC3315021

[13] Burrell JR, Halliday GM, Kril JJ, Ittner LM, Götz J, Kiernan MC, et al The frontotemporal dementia-motor neuron disease continuum. The Lancet. 2016 3 14. 10.1016/S0140-6736(16)00737-6 26987909

[14] Elamin M, Pender N, Hardiman O, Abrahams S. Social cognition in neurodegenerative disorders: a systematic review. J Neurol Neurosurg Psychiatry 2012; 83:1071–1079. doi: 10.1136/jnnp-2012-302817 22869923 10.1136/jnnp-2012-302817

[15] Jelsone-Swain L, Persad C, Burkard D, Welsh RC. Action Processing and Mirror Neuron Function in Patients with Amyotrophic Lateral Sclerosis: An fMRI Study. PLoS ONE 2105; 10(4): doi: 10.1371/journal.pone.0119862 10.1371/journal.pone.0119862 PMC4401664 25885533

[16] Mason SL, Zhang J, Begeti F, Valle Guzman N, Lazar AS, Rowe JB, et al The role of the amygdala during emotional processing huntington’s disease: from pre-manifest to late stage disease. Neuropsychologia 2015;70:80–89. doi: 10.1016/j.neuropsychologia.2015.02.017 25700742 10.1016/j.neuropsychologia.2015.02.017 PMC4415907

[17] Heitz C, Vogt N, Cretin B, Philippi N, Jung B, Phillipps C, et al Cognitive and affective theory of mind in Lewy body dementia: A preliminary study. Revue Neurologique 2015;171(4):373–381. doi: 10.1016/j.neurol.2015.02.010 25847396 10.1016/j.neurol.2015.02.010

[18] De Ferrari AR, Lagravinese G, Pelosin E, Pardini M, Serrati C, Abbruzzese G, et al Freezing of gait and affective theory of mind in Parkinson disease, Parkinsonism and Related Disorders (2015), doi: doi10.1016/j.parkreldis.2015.02.023 10.1016/j.parkreldis.2015.02.023 25772323

[19] Boucher O, Rouleau I, Lassonde M, Lepore F, Bouthillier A, Nguyen DK. Social information processing following resection of the insular cortex. Neuropsychologia 2015;71: 1–10. doi: 10.1016/j.neuropsychologia.2015.03.008 25770480 10.1016/j.neuropsychologia.2015.03.008

[20] Gawęda L, Krężolek M, Olbryś J, Turska A, Kokoszka A. Decreasing self-reported cognitive biases and increasing clinical insight through meta-cognitive training in patients with chronic schizophrenia. Journal of behavior therapy and experimental psychiatry 2015;48: 98–104. doi: 10.1016/j.jbtep.2015.02.002 25775947 10.1016/j.jbtep.2015.02.002

[21] Nentjes L, Bernstein D, Arntz A, van Breukelen G, Slaats M. Examining the influence of psychopathy, hostility biases, and automatic processing on criminal offenders’ Theory of Mind. International journal of law and psychiatry 2015; 38: 92–99. doi: 10.1016/j.ijlp.2015.01.012 25655653 10.1016/j.ijlp.2015.01.012

[22] Levy NK, Milgram N. Cognitive contributions to theory of mind ability in children with a traumatic head injury, Child Neuropsychology: A Journal on Normal and Abnormal Development in Childhood and Adolescence 2014;: . , doi: 10.1080/09297049.2014.985642 10.1080/09297049.2014.985642 25495376

[23] Cassels TG, Birch SAJ. Comparisons of an Open-Ended vs. Forced-Choice ‘Mind Reading’ Task: Implications for Measuring Perspective-Taking and Emotion Recognition. PLoS ONE 2014; 9(12): doi: 10.1371/journal.pone.0093653 10.1371/journal.pone.0093653 PMC4256375 25474645

[24] El Haj M, Raffard S Gély-Nargeot M. Destination memory and cognitive theory of mind in normal ageing. Memory 2015;: . doi: 10.1080/09658211.2015.1021257 10.1080/09658211.2015.1021257 25768052

[25] Khorashad BS, Baron-Cohen S, Roshan GM, Kazemian M, Khazai L, Aghili Z, et al The “Reading the Mind in the Eyes” Test: Investigation of Psychometric Properties and Test-Retest Reliability of the Persian Version. J Autism Dev Disord 2015; 1–16 doi: 10.1007/s10803-015-2427-4 25832800 10.1007/s10803-015-2427-4

[26] Lodder GMA, Scholte RHJ, Goossens L, Engels RCME, Verhagen M. Loneliness and the social monitoring system: emotion recognition and eye gaze in real-life conversation. JOURNAL 2015 doi: 10.1111/bjop.12131 10.1111/bjop.12131 25854912

[27] Fernandez-Abascal EG, Cabello R, Fernandez-Berrocal P, Baron-Cohen S. Test-retest reliability of the ‘Reading the Mind in the Eyes’ test: A one-year follow-up study. Molecular Autism 2013; 4, 33 doi: 10.1186/2040-2392-4-33 24020728 10.1186/2040-2392-4-33 PMC3848772

[28] Ahmed FS, Stephen ML. Executive function mechanisms of theory of mind. Journal of Autism and Developmental Disorders 2011; 41(5): 667–678. doi: 10.1007/s10803-010-1087-7 20811770 10.1007/s10803-010-1087-7

[29] Phukan J, Elamin M, Bede P, Jordan N, Gallagher L, Byrne S, et al The syndrome of cognitive impairment in amyotrophic lateral sclerosis: a population-based study. J Neurol Neurosurg Psychiatry 2012; 83:102–108. doi: 10.1136/jnnp-2011-300188 21836033 10.1136/jnnp-2011-300188

[30] Strong MJ, Grace GM, Freedman M, Lomen-Hoerth C, Woolley S, Goldstein L, et al Consensus criteria for the diagnosis of frontotemporal cognitive and behavioural syndromes in amyotrophic lateral sclerosis. Amyotroph Lateral Scler 2009;10:131e46. 19462523 10.1080/17482960802654364

[31] O’Toole O, Traynor BJ, Brennan P, Sheehan C, Frost E, Corr B, et al Epidemiology and clinical features of amyotrophic lateral sclerosis in Ireland between 1995 and 2004. J Neurol Neurosurg Psychiatry 2008;79:30–32. 17634215 10.1136/jnnp.2007.117788

[32] Brooks BR, Miller RG, Swash M, Munsat TL. El Escorial revisited: revised criteria for the diagnosis of amyotrophic lateral sclerosis. Amyotroph Lateral Scler Other Motor Neuron Disord 2000;1:293–299. 11464847 10.1080/146608200300079536

[33] Holdnack H. A. (2001). Wechsler Test of Adult Reading: WTAR. San Antonio: The Psychological Corp.

[34] Raven J, Raven JC, Court JH. (1998). Manual for Raven's Progressive Matrices and Vocabulary Scales Section 2: The Coloured Progressive Matrices. San Antonio, TX: Harcourt Assessment.

[35] Linda C, Algina J. (2006). Introduction to classical and modern test theory Mason, OH: Cengage Learning.

[36] Kelley TL. The selection of upper and lower groups for the validation of test items. Journal of Educational Psychology 1939;30:17–24.

